# The Prognosis in Renal Cases

**Published:** 1921-01-29

**Authors:** 


### EVERYDAY MEDICAL PROBLEMS.

### I.—The Prognosis in Renal Cases.

There are few more puzzling problems likely to face the medical practitioner than those connected with the ability of a patient's kidneys to perform the work required of them and the span of years over which in any particular person the renal function may be expected to continue adequate. The well-known remark that a man is as old as his arteries may be to a large extent true, but in this respect the kidneys also have a strong claim to attention. Quite recently the medical journals published a somewhat technical discussion of the subject, mainly from the insurance companies' standpoint, and although one hesitates to conjecture that some of the reasoning was a little "above the heads" of many busy practitioners, yet there is probably advantage to be derived from a review of some simpler essentials.

### The Meaning of Albuminuria.

The first point to remember is that the presence of albumin in the urine, at one time regarded as indicative of nephritis, is now recognised to occur in many circumstances without the existence of any serious organic change in the kidneys. To summarise, it may be accepted that the occurrence of a physiological albuminuria can follow muscular work, food very rich in albumin, violent emotions, cold bathing and severe dyspepsia. The presence of this albumin may represent some transient change in the kidney cells, but it is of no serious significance for the future. More interesting and more controversial is *orthostatic albuminuria*, sometimes occurring in male adolescents, and appearing intermittently. Often the condition is discovered quite accidentally. The patients are generally those of markedly neurotic tendency and unstable vasomotor system. As this name implies, even change to the upright position may sufficiently influence the renal circulation to produce transudation of albumin. But according to Goodhart, who recorded 250 cases and followed them carefully, albuminuria of the adolescent, to which all such instances are probably mechanically allied, has no bad effect either on health or on the duration of life. Within reasonable limits these people should not be excluded from the advantages of life insurance. A certain number tend to develop a gradual increase in arterial tension, indicative of slow changes in the kidney tissue, but in practically all these, the original albuminuria is noticeably severe, and the statement still stands that the case of a young man who shows a sound general system and an otherwise normal urinary excretion, is not to be viewed seriously.

It is unnecessary to detail the renal effects of high temperature. They are commonly seen in malaria, diphtheria, typhoid, etc. The results of a febrile disease may vary from the mildest albuminuria to a toxic or an acute nephritis, and the prognosis depends entirely as to whether, after the immediate recovery, any permanent damage to the kidney remains. Passage of albumin from the blood to the urine *viâ* the renal cells occurs in a variety of conditions where the blood itself has undergone degenerative changes as in purpura, leukæmia, profound anæmias, etc., and the transient albuminuria of pregnancy is probably in the same class. And lastly, toxins temporarily present in the blood—such as lead, mercury, the virus of syphilis—may affect the renal tissue with a corresponding result.

This list, then, includes the group of conditions in which true serum albumen may be passed in the urine and yet not necessarily convey a sinister import as to the patient's renal condition. But, it must not be forgotten that other proteins, *not* serum albumin, may be present. These include albumose, peptone, globulin, etc., and they are generally of but slight clinical significance, for they may be found whenever protein materials are undergoing autolysis in the system, as in resolving pneumonias or even during involution of the uterus. Cellular *débris* in the urine, leucocytes and small amounts of blood itself, also naturally give the urine a variable protein content, but beyond reminding the practitioner that small quantities of these cellular structures mixed with the urine can thus mislead him, there is little need further to follow their pathology. The microscope will always decide. Bence-Jones' proteosuria, as pathognomonic of multiple myelomata, is still a classic of the laboratory.

### True Renal Disease.

On the subject of true nephritis and its picture the text-books are amply explicit. The meaning of renal tube casts in the urine depends entirely on the type of material from which the casts are made (simple exudate or broken-down renal tissue). The simple hyaline type is unimportant enough, and, as in this note, one is touching chiefly spurious threatenings of danger ahead, it may be well simply to quote Osler's summary of the situation. "A trace of albumen in a man over forty, with or without a few hyaline casts, is not of much significance, except as an indication that his kidneys, like his hair, are beginning to turn 'grey' with age. In many instances the discovery is of positive advantage, as the man is made to realise, perhaps for the first time, that he has been living carelessly. The question was discussed from this standpoint in a paper with the paradoxical title 'On the advantages of a Trace of Albumen and a few Tube-casts in the Urine of Men over Fifty Years of Age'"!

### Methods of Estimation.

But supposing that a particular renal case does not fall into any of these few relatively harmless categories, and the practitioner finds that the kidneys' efficiency probably falls short of 100 per cent., by what relatively accurate means can he determine its real degree? In making any selection or summary there is risk of incurring the criticism

**Everyday Medical Problems**—(*continued*).

of one or other pathological school, so essentials only must be adhered to. The possible investigations may be divided into two classes: (*a*) Those to determine whether excretion is defective; (*b*) those to ascertain whether there is undue retention in the blood. That is, either the urine or the blood may be the medium for estimations. On the whole, those upon the latter are probably the more accurate and valuable.

A useful summary of the information such tests may give is provided by Dr. O. C. Gruner as below:

They may indicate: (1) The existence of any renal disturbance at all; (2) determine the extent of impairment for diagnostic as well as prognostic purposes; (3) assessment of the danger to life for some other disease whose recovery depends on a sound kidney; (4) a method of suitable diet; (5) a preliminary guide to undertaking operation requiring seven to ten days' observation.

Somewhere in this list the practitioner can place practically every renal circumstance in which he may wish to decide. Technically the actual tests are far beyond the scope of this note, but in conclusion it may be well to indicate in outline one or two of the simpler ones which the pathologist may be requested to perform and that with the best hope that results will justify the trouble taken.

In the first place, concentrations of urea or creatinine in the blood are valuable indications. About 20 mg. of urea nitrogen per 100 cc. is the upper normal limit and Maclean and Wesselow consider that in early nephritis this blood-urea estimate gives probably the best and simplest indication of the functional state of the kidneys. Also the injection of the dye *Phenol-sulphonephthalein* and experimental observation of the rate of its excretion into the bladder is an excellent method of detecting impairment of renal function. The test does not differentiate between the actual types of nephritis, but it appears to show whether the kidney is reasonably sound, which may indeed be safely assumed if forty to fifty per cent. of the dye is excreted within one hour. A further simple test upon the urine itself consists in giving urea by the mouth and estimating the urinary urea at intervals of half an hour. It is generally found that these results run parallel with the phthalein test described above. Another straightforward test, and also one of great value in conjunction with the urea estimate, is accurate determination of the diastatic power of the urine. With these methods it is possible to gain considerable insight to the renal efficiency and apart from their relative reliability they are mentioned as being readily within the scope of the ordinary clinical laboratory to which a practitioner can appeal. The reasons that may make it advisable for him to do so have already been outlined.

